# Urine culture doubtful in determining etiology of diffuse symptoms among elderly individuals: a cross-sectional study of 32 nursing homes

**DOI:** 10.1186/1471-2296-12-36

**Published:** 2011-05-19

**Authors:** Pär-Daniel Sundvall, Peter Ulleryd, Ronny K Gunnarsson

**Affiliations:** 1 Sandared Primary Health Care Centre, Strandvägen 11, 518 32 Sandared, Sweden; 2 Research and development unit, Primary Health Care in Southern Älvsborg County, Sven Eriksonsplatsen 4, 503 38 Borås, Sweden; 3 Department of Public Health and Community Medicine, Institute of Medicine, The Sahlgrenska Academy, University of Gothenburg, Box 454, 405 30 Göteborg, Sweden; 4 Department of Communicable Disease Control and Prevention, Region Västra Götaland, Institute of Biomedicine, The Sahlgrenska Academy, Kaserntorget 11 B, 411 18 Göteborg, Sweden

**Keywords:** Urinary Tract Infections, Homes for the Aged, Nursing Homes, Bacteriuria, Predictive Value of Tests

## Abstract

**Background:**

The high prevalence of bacteriuria in elderly individuals makes it difficult to know if a new symptom is related to bacteria in the urine. There are different views concerning this relationship and bacteriuria often leads to antibiotic treatments. The aim of this study was to investigate the relationship between bacteria in the urine and new or increased restlessness, fatigue, confusion, aggressiveness, not being herself/himself, dysuria, urgency and fever in individuals at nursing homes for elderly when statistically considering the high prevalence of asymptomatic bacteriuria in this population.

**Methods:**

In this cross-sectional study symptoms were registered and voided urine specimens were collected for urinary cultures from 651 elderly individuals. Logistic regressions were performed to evaluate the statistical correlation between bacteriuria and presence of a symptom at group level. To estimate the clinical relevance of statistical correlations at group level positive and negative etiological predictive values (EPV) were calculated.

**Results:**

Logistic regression indicated some correlations at group level. Aside from *Escherichia coli *in the urine and not being herself/himself existing at least one month, but less than three months, EPV indicated no clinically useful correlation between any symptoms in this study and findings of bacteriuria.

**Conclusions:**

Urinary cultures provide little or no useful information when evaluating diffuse symptoms among elderly residents of nursing homes. Either common urinary tract pathogens are irrelevant, or urine culture is an inappropriate test.

## Background

The prevalence of bacteriuria in elderly residents of nursing homes without indwelling urinary catheters varies from 25% to 50% for women and 15% to 40% for men [1,2]. Bacteriuria may represent a symptomatic urinary tract infection (UTI) or an asymptomatic bacteriuria (ABU). The latter should not be treated with antibiotics [3-7]. When a patient with a new or increased symptom also has bacteriuria, the high prevalence of ABU makes it difficult to know if the bacteriuria is just an ABU not influencing symptoms, or if the bacteriuria is an UTI that may have caused symptoms [8-10]. It has been estimated that if a urinary culture is positive in an institutionalized febrile elderly individual without an indwelling urinary catheter and with no local findings, less than 10% of such episodes are attributable to a UTI [11]. Furthermore, the combination of bacteriuria and pyuria can not differentiate between UTI and ABU in individuals at nursing homes for elderly individuals [12]. The problem of differentiation between UTI and ABU is even more difficult for diffuse non-specific symptoms such as restlessness, fatigue, confusion, aggressiveness or not being herself/himself. These symptoms can have many other causes than UTI [13]. There are different views concerning the relationship between various non-specific symptoms and UTI [14-19].

Change in mental status (lethargy, disorientation, increased or new onset of confusion and delirium) was the most commonly reported symptom for suspecting UTI [20]. Non-specific symptoms and signs are important factors in antibiotic prescription for ABU in elderly institutionalized people despite lack of evidence [21]. Despite evidence that ABU should not be treated, suspected UTI remains the most frequent reason for prescribing antibiotics in nursing homes for the elderly [22]. This probably reflects the diagnostic uncertainty of UTI among nursing homes for the elderly [10,23].

Elderly people are more likely to experience adverse drug events of antibiotic treatment [24]. However the main reason for avoiding unnecessary antibiotic treatment is increasing antibiotic resistance. Thus it is important to study the role of bacteriuria in relation to non-specific symptoms.

The aim of this study was to investigate the relationship between bacteria in the urine and new or increased restlessness, fatigue, confusion, aggressiveness, not being herself/himself, dysuria, urgency and fever in individuals at nursing homes for elderly when statistically considering the high prevalence of asymptomatic bacteriuria in this population.

## Methods

Eventual symptoms were registered in the study protocol and a single voided specimen of urine was collected. A urine dipstick was analyzed and urine cultured from elderly individuals at nursing homes during a four-week period in the first months of 2003. The nursing homes were located in southwestern Sweden. The study was approved by the ethical committee of Gothenburg University. The data was collected along with another study evaluating dipstick urinalysis among elderly residents [2].

### Study population

Individuals at the nursing homes, regardless of symptoms, were asked to participate. One single urine specimen was collected from each included, permanent elderly resident of 102 wards in 32 nursing homes. The following inclusion criteria were applied:

• Permanent residence in nursing homes for elderly (regardless of gender).

• No indwelling urinary catheter (as they always become bacteriuric).

• Sufficiently continent to leave a voided urinary specimen (obtaining urinary specimens by catheterizing these individual would not be representative of clinical practice and furthermore unethical).

• Present at the nursing home for elderly when the study took place.

• No ongoing dialysis.

• Participation approval.

The following exclusion criterion was used:

• If the individual did not agree to participate or wished to discontinue study participation.

For individuals with dementia the same inclusion/exclusion criteria were applied. However substantial incontinence among these individuals with dementia sometimes made it impossible to obtain a voided specimen of urine.

### Statement of consent

Individuals were informed about the study verbally and in written text. Informed approval for participation in the study was collected from decision-capable individuals that chose to participate in the study.

A considerable number of the participants, however, consisted of individuals with varying degrees of dementia. If the individual had no possibility to comprehend the meaning of the study information and thereby had a reduced decision-capability, these individuals only participated as long as they did not oppose participation and under the condition that a relative/appointed representative did not oppose their participation after having taken part of the study information. Thus, in case of dementia, where the individual did not understand the provided information, a sample was taken only if permission was granted. This procedure was approved by the ethical committee, University of Gothenburg.

### Study protocol

The attending nurse at the nursing home registered, in a study protocol, once for each individual according to carefully provided instructions, if any of the following symptoms had appeared or increased: restlessness, fatigue, confusion, aggressiveness or not being herself/himself, dysuria, urgency and fever. Depending on how long the symptoms had persisted or increased, they were divided into the following three groups: less than one week, more then one week but less than one month, more than one month but less than three months. A symptom was not registered if it had persisted unchanged longer than three months. The study protocol included whether the individual had ongoing or previous antibiotic treatment within the last month.

### Laboratory tests

The personnel at the nursing home were instructed to collect voided urine specimens with as long a bladder incubation time as possible, preferably a mid-stream morning sample. Then, dipstick urinalysis was carried out at the nursing home. The attending nurses at the nursing home were carefully instructed to register presence or absence of symptoms in the study protocol before performing dipstick analysis and taking a urinary culture. Thus the evaluation of symptoms was not influenced by the results of the urine tests.

Immediately after the dipstick readings the urine samples were chilled before transport to the microbiological laboratory in Borås where urine specimens were cultured. The samples usually reached the laboratory within 24 hours.

Using sterile inoculating loops the microbiology laboratory fractionated 10 μl urine on the surfaces of two plates; a cystine-lactose-electrolyte deficient agar (CLED) and a Columbia blood agar base. Plates were incubated overnight (minimum 15 h) at 35-37°C. CLED plates were incubated in air and Columbia plates were incubated in 5% CO_2_. The latter was further incubated for 24 hours if no growth occured after the first incubation.

Growth of potentially pathogenic bacteria was usually considered positive if the number of colony-forming units per liter (CFU/mL) was ≥10^5^. However, at signs of possible UTI such as positive nitrite dipstick, leukocyte esterase dipstick >1, fever, frequency, urgency or dysuria, the cut-off point was ≥10^3 ^for patients with growth of *Escherichia coli *and male patients with *Klebsiella *species and *Enterococcus faecalis*. For symptomatic women harboring the two latter species the cut-off level was ≥10^4^.

### Statistical analysis

Depending on how long symptoms had persisted, they were divided into the following three groups; less than one week, less than one month, and less than three months. To evaluate the statistical correlation between bacteriuria and presence of a symptom at group level logistic regressions were performed. The symptom was used as dependent variable and the outcome of the urinary culture, age and sex as independent variables. One regression was made for each symptom and duration. One regression was also made for each symptom merging time intervals to all occurring within three months.

To estimate clinical relevance of statistical correlations, the positive and negative etiological predictive value (EPV) [25] was calculated to evaluate the probability for a positive/negative culture to rule in or out that a symptom in a single individual is associated with a bacterial finding. These predictive values were considered clinically useful if their point estimate was ≥ 75% with a lower 95% confidence interval ≥ 50%.

Logistic regression is a more sensitive statistical method than EPV. It is common that regression shows statistical significance while positive EPV does not show that a positive test outcome is clinically relevant. The opposite can never occur. Negative EPV is less affected by asymptomatic carriers and will often be high in case of a negative test.

Epi Info version 3.3.2 (Windows version) (CDC, Atlanta, USA) was used for storing data and for logistic regression. EPV with confidence intervals was calculated using the EPV-Calculator version 1.12 [26].

## Results

**Figure 1:**
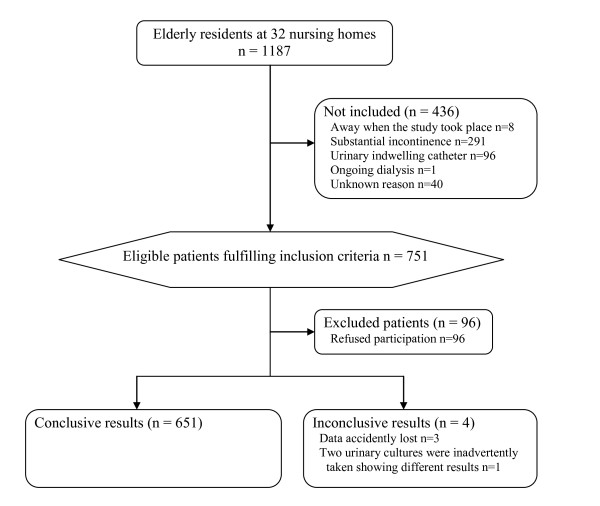
Participents Flow Chart

Of 1187 individuals in 32 nursing homes (Figure 1) 751 fulfilled the inclusion criteria. 655 (87%) of these accepted participation (Figure 1). 651 individuals provided useful samples, 482 (74%) were women and 169 (26%) men. Women's ages (mean 86 years, SD 7.4, range 46-102) were slightly higher than men's (mean 82 years, SD 7.8, range 54-99) (p < 10^-4^).

100/651 (15%) had diabetes mellitus. When the urine specimen was collected, 26/651 (4.0%) were in antibiotic treatment. Another 61/651 (9.4%) were not in antibiotic treatment when the urine specimen was collected but had had antibiotic treatment during the previous month. Antibiotic treatment history was, however, unknown for 12/651 (1.8%).

### The most common bacterium

In this study 207/651 (32%) urine cultures showed growth of a potentially uropathogenic bacterial species. The most common finding was *E. coli *(143 = 22%), *Klebsiella *spp. (25 = 3.8%) and *E. faecalis *(17 = 2.6%). Other species had very low prevalence (≤0.8% for each species).

### Prevalence of symptoms

Fatigue, restlessness and confusion were the most common symptoms (Table 1-2). Combined symptoms were uncommon. The four most prevalent combined symptoms were restlessness and fatigue 11/651 (1.7%), fatigue and confusion 7/651 (1.1%), fatigue and urgency 6/651 (0.92%) and restlessness and confusion 6/651 (0.92%).

**Table 1:** Odds ratios and probabilities that findings of *Escherichia coli *in the urine^1 ^are associated with symptoms.

		*Statistical correlation between bacteriuria and presence of symptom*		*Probability (%) for positive/negative culture to rule in/rule out that symptom is associated with bacterial finding*
Symptoms	Prevalence^2^	Odds ratio (95% CI)	p-value	Positive/negative etiologic predictive value^3^
Restlessness	9.1%	1.4 (0.76-2.6)	0.28	34 (0-72)/99 (94-100)
Fatigue	12%	1.7 (0.99-2.9)	0.057	40 (0-73)/98 (94-100)
Confusion	7.5%	1.8 (0.96-3.6)	0.067	46 (0-79)/98 (92-100)
Aggressiveness	4.3%	2.3 (0.96-5.6)	0.063	44 (0-82)/98 (90-100)
Not being herself/himself	2.3%	4,4 (1.5-13)	0.0080	79 (0-98)/90 (50-100)
Dysuria	1.8%	1.6 (0.47-5.7)	0.44	46 (0-90)/98 (82-100)
Urgency	5.5%	1.3 (0.58-2.9)	0.52	17 (0-70)/99 (95-100)
Fever	0.31%	0 (0-∞)	0.97	---^4^

^1^Escherichia coli was found in the urine in 143 of 651 urine samples.

^2^Appearance or increase of symptom or signs within the last three months in the studied population. All patients with fever acquired this within the last week.

^3^Etiologic predictive value (EPV) is a statistical method used to evaluate the clinical usefulness of dichotomous diagnostic tests. It estimates predictive values when there is no proper gold standard for comparison. EPVs are presented in this table with 95% confidence interval within parenthesis. To calculate the EPV the sensitivity of a urinary culture to detect presence of bacteriuria was estimated at 90%.

^4^The proportion of positive tests among individuals without the specified symptom or sign exceeds the proportion of positive tests among individuals with the specified symptom or sign. EPV cannot be calculated in these circumstances.

### Correlation between bacteriuria and symptoms

The prevalence of bacteriuria for each symptom was: restlessness 39%, fatigue 41%, confusion 45%, aggressiveness 39%, not being herself/himself 60%, dysuria 42%, urgency 42% and fever 50%.

Not being herself/himself that occurred within 3 months correlated with findings of *E. coli *(Table 1) or any bacteria (Table 2). Fever acquired within 1 week correlated with findings of *Klebsiella *spp. (odds ratio 45 with 95% CI 2.0-980, p = 0.016). Confusion or fatigue that occurred or changed within 3 months correlated with findings of any bacteria (Table 2).

**Table 2:** Odds ratios and probabilities that findings of any bacteria in the urine^1 ^are associated with symptoms.

		*Statistical correlation between bacteriuria and presence of symptom*		*Probability (%) for positive/negative culture to rule in/rule out that symptom is associated with bacterial finding*
Symptoms	Prevalence^2^	Odds ratio (95% CI)	p-value	Positive/negative etiologic predictive value^3^
Restlessness	9.1%	1.3 (0.76-2.4)	0.31	31 (0-71)/98 (91-100)
Fatigue	12%	1.7 (1.0-2.7)	0.046	40 (0-72)/97 (91-100)
Confusion	7.5%	1.9 (1.0-3.5)	0.044	48 (0-80)/96 (86-100)
Aggressiveness	4.3%	1.7 (0.77-3.9)	0.19	31 (0-78)/98 (87-100)
Not being herself/himself	2.3%	3.3 (1.1-9.9)	0.030	74 (0-99)/88 (17-100)
Dysuria	1.8%	1.4 (0.43-4.6)	0.57	37 (0-90)/97 (72-100)
Urgency	5.5%	1.7 (0.85-3.5)	0.13	38 (0-79)/97 (87-100)
Fever	0.31%	3.2 (0.17-61)	0.44	56 (0-100)/94 (0-100)

^1^Any bacteria were found in the urine in 207 of 651 urine samples.

^2^Appearance or increase of symptom or signs within the last three months in the studied population. All patients with fever acquired this within the last week.

^3^Etiologic predictive value (EPV) is a statistical method used to evaluate the clinical usefulness of dichotomous diagnostic tests. It estimates predictive values when there is no proper gold standard for comparison. EPVs are presented in this table with 95% confidence interval within parenthesis. To calculate the EPV the sensitivity of a urinary culture to detect presence of bacteriuria was estimated at 90%.

If duration of symptoms includes all patients with new or changed symptoms within 3 months, as in Table 1-2, the etiologic predictive values suggest that none of these statistical findings had any clinical relevance (Table 1-2). However, when analyzing duration of symptoms separately EPV showed that in a patient where not being herself/himself for the preceding period of >1 months and ≤3 months growth of *E. coli *was with 96% (51-100%) probability associated to this symptom. No other subset of duration showed any clinically relevant EPV.

## Discussion

It is common that a patient at a nursing home for elderly individuals often has diffuse symptoms such as restlessness, fatigue, confusion, aggressiveness or not being herself/himself. This study suggests that a urinary culture is of little or no value in clarifying the etiology of these symptoms.

### Strength and limitations of the study

Are the bacteria in the urine only to be considered an insignificant finding (ABU) or is a lower UTI causing the new symptom? Ordinary predictive values cannot be calculated since there is no gold standard for this situation. Thus, it's impossible to know whether a positive urinary culture in a patient with a new symptom is related to the new symptom or not as the prevalence of asymptomatic bacteriuria among individuals at nursing homes for the elderly varies between 25% and 50% for women and 15% and 40% for men. Using a prospective study following individuals over time and recording new symptoms does not solve the problem of a missing gold standard. A possible way to solve this would be a large randomized controlled trial (RCT). However, an RCT in a large population of fragile elderly individuals is not easy to perform. Another possibility to bypass the problem of a missing gold standard is to use the new statistical method of EPV. The EPV, which is used in this study, produces predictive values statistically considering the presence of insignificant findings, i.e. asymptomatic carriers, in the absence of a gold standard. EPV can only be calculated if we analyze a urine sample in both symptomatic and asymptomatic individuals.

In this study we obtained urine specimens and study protocols from 55% (651/1187) of individuals registered at the nursing homes. This may appear low but is similar to previously published studies in nursing homes for the elderly [8]. The main reason for not participating in this study was substantial urinary incontinence, often combined with dementia. Obtaining a urine specimen from these individuals would have required the use of a catheter which is not routine in clinical practice for elderly at nursing homes and would, therefore, not have been representative of clinical practice. Individuals with an indwelling urinary catheter were excluded as they always become colonized by bacteria that are sometimes of different species compared to those without catheters [1]. Only 13% (96/751) refused participation which may be considered acceptable.

The symptoms investigated in this study are common causes for antibiotic treatment despite clear evidence that they are associated with lower UTI. Symptoms were not diagnosed using validated questionnaires or other validated instruments. Instead, we used the labels used by most nurses for describing patients' symptoms when consulting a physician. Thus, labeling patients with, for example, confusion and not being herself/himself overlap. The reason for using labels commonly used by nurses is that nurses play a central role in both the ordering of urine cultures and the decision to prescribe antibiotics [21]. Thus, a more constructive way to look upon this is that the present study does not evaluate objective symptoms but labels of symptoms commonly used by nurses and presented to the physician.

Several symptoms may coexist. This could be a potential confounding factor that should have been addressed in the regression analyses. However, even the most frequent combinations of symptoms were rare.

Three time intervals for duration of symptoms were chosen to investigate if more detailed information concerning duration of symptoms was of great importance. It turned out that in most situations more detailed information on different durations, other than duration ≤3 months, was not of clinical importance.

Only 4.0% (26/651) had ongoing antibiotic treatment, regardless of antibiotic or underlying infection. Thus for those individuals that had antibiotics affecting urinary pathogens, no urinary bacteria growth may have been expected. Due to the low prevalence of ongoing antibiotic treatment this effect was considered low.

Procedures utilizing presence of symptoms or outcome of prior dipstick testing to influence the decision of cut-off levels for CFU in urine culture may enhance the diagnostic procedure [27]. These procedures are common in microbiologic laboratories in Sweden. Thus, the common present procedure for urine culture was used without modification to be representative of ordinary clinical practice. It should be mentioned that no diffuse symptoms such as restlessness, fatigue, confusion, aggressiveness or not being herself/himself influenced decisions on cut-off levels for CFU in the urine cultures.

### Findings of or absence of bacteriuria

While the results of the logistic regressions are of interest at group level, these results are of limited value to the physician making a clinical decision for a single patient. The EPV however, is of greater clinical importance to the physician as a positive EPV describes the probability of findings of bacteriuria to be associated with symptoms. Since EPV considers asymptomatic carriers it provides an estimate of the clinical relevance of bacteriuria. It is interesting to note that none of the estimates of positive EPV showed that bacteriuria had any clinical relevance except for findings of *E. coli *in patients not being herself/himself since at least one month, but less than three months. Thus, with most symptoms, findings of bacteria in the urine do not add etiologic information and physicians must investigate the possibility of other explanations than lower UTI.

Considering every time interval for duration it turned out that all symptoms, except dysuria and urgency, had at some time interval a statistical correlation at a group level between symptoms and findings of bacteria in the urine. ABU increases when an individual's general health declines. Therefore the association between some diffuse symptoms and bacteriuria might be explained by the fact that when an individual's health declines they develop more diffuse symptoms and at the same time are more prone to ABU. Thus, it might not be causality between bacteriuria and symptoms. To determine this, a randomized controlled trial with antibiotic treatment in patients not being herself/himself is needed.

It is not surprising to find that absence of bacteriuria usually indicates that the symptom is not associated with bacteriuria (table 1-2).

### Urgency or dysuria

It is remarkable that there was no correlation between either urgency or dysuria and bacteriuria. This might be explained by the fact that there are many other causes of urgency and dysuria than UTI among individuals living at nursing homes for elderly [28-30]. Thus UTI may be only a minor cause of these symptoms.

## Conclusions

The results of this study can be considered valid in ordinary clinical practice in developed countries when a physician is evaluating elderly individuals at nursing homes with new or increased diffuse symptoms. Except for *E. coli *in the urine and not being herself/himself of at least one month but less than three months, EPV indicated no correlation between symptoms in this study and findings of bacteriuria. Thus, with this possible exception (to be confirmed by a randomized clinical trial) it is not useful to perform urinary cultures when evaluating these symptoms among elderly residents. These results can be interpreted as either that common urinary tract pathogens are of no relevance, or a urine culture alone is inappropriate, for evaluating this specific situation. Searching for a new biomedical test to supplement or even replace urine culture seems clinically important for this particular situation.

## Competing interests

The authors declare that they have no competing interests.

## Authors' contributions

PDS and RG designed the study. PDS carried out the data collection. PDS, PU and RG performed the analyses and participated in writing the manuscript. All authors reviewed and approved the final draft of the paper.

## Pre-publication history

The pre-publication history for this paper can be accessed here:

http://www.biomedcentral.com/1471-2296/12/36/prepub

## References

[B1] Nicolle LE Urinary tract infections in long-term-care facilities Infect Control Hosp Epidemiol 2001 22 3 167 175 10.1086/501886 11310697

[B2] Sundvall PD Gunnarsson RK Evaluation of dipstick analysis among elderly residents to detect bacteriuria: a cross-sectional study in 32 nursing homes BMC Geriatr 2009 9 32 10.1186/1471-2318-9-32 19635163 PMC2724370

[B3] Nicolle LE Bjornson J Harding GK MacDonell JA Bacteriuria in elderly institutionalized men N Engl J Med 1983 74 4 1420 1425 10.1056/NEJM198312083092304 6633618

[B4] Nicolle LE Mayhew WJ Bryan L Prospective randomized comparison of therapy and no therapy for asymptomatic bacteriuria in institutionalized elderly women Am J Med 1987 83 1 27 33 3300325 10.1016/0002-9343(87)90493-1

[B5] Harding GK Zhanel GG Nicolle LE Cheang M Antimicrobial treatment in diabetic women with asymptomatic bacteriuria N Engl J Med 2002 347 20 1576 1583 10.1056/NEJMoa021042 12432044

[B6] Ouslander JG Schapira M Schnelle JF Uman G Fingold S Tuico E Nigam JG Does eradicating bacteriuria affect the severity of chronic urinary incontinence in nursing home residents? Ann Intern Med 1995 122 10 749 754 7717597 10.7326/0003-4819-122-10-199505150-00003

[B7] Nicolle LE Bradley S Colgan R Rice JC Schaeffer A Hooton TM Infectious Diseases Society of America guidelines for the diagnosis and treatment of asymptomatic bacteriuria in adults Clin Infect Dis 2005 40 5 643 654 10.1086/427507 15714408

[B8] Hedin K Petersson C Widebäck K Kahlmeter G Mölstad S Asymptomatic bacteriuria in a population of elderly in municipal institutional care Scand J Prim Health Care 2002 20 3 166 168 10.1080/028134302760234627 12389754

[B9] Rodhe N Löfgren S Strindhall J Matussek A Mölstad S Cytokines in urine in elderly subjects with acute cystitis and asymptomatic bacteriuria Scand J Prim Health Care 2009 27 2 74 79 10.1080/02813430902757634 19247873 PMC3410465

[B10] Nicolle LE Urinary tract infection in long-term-care facility residents Clin Infect Dis 2000 31 3 757 761 10.1086/313996 11017826

[B11] Orr PH Nicolle LE Duckworth H Brunka J Kennedy J Murray D Harding GK Febrile urinary infection in the institutionalized elderly Am J Med 1996 100 1 71 77 10.1016/S0002-9343(96)90014-5 8579090

[B12] Nicolle LE Urinary infections in the elderly: symptomatic of asymptomatic? Int J Antimicrob Agents 1999 11 3-4 265 268 10.1016/S0924-8579(99)00028-X 10394981

[B13] Bostwick JM The many faces of confusion. Timing and collateral history often hold the key to diagnosis Postgrad Med 2000 108 6 60 2 65-6 71-2 11098259 10.3810/pgm.2000.11.1288

[B14] Nicolle LE Henderson E Bjornson J McIntyre M Harding GK MacDonell JA The association of bacteriuria with resident characteristics and survival in elderly institutionalized men Ann Intern Med 1987 106 5 682 686 3565965 10.7326/0003-4819-106-5-682

[B15] Juthani-Mehta M Quagliarello V Perrelli E Towle V Van Ness PH Tinetti M Clinical features to identify urinary tract infection in nursing home residents: a cohort study J Am Geriatr Soc 2009 57 6 963 970 10.1111/j.1532-5415.2009.02227.x 19490243 PMC2692075

[B16] Eriksson S Strandberg S Gustafson Y Lundin-Olsson L Circumstances surrounding falls in patients with dementia in a psychogeriatric ward Arch Gerontol Geriatr 2009 49 1 80 87 10.1016/j.archger.2008.05.005 18635273

[B17] Juthani-Mehta M Tinetti M Perrelli E Towle V Van Ness PH Quagliarello V Diagnostic accuracy of criteria for urinary tract infection in a cohort of nursing home residents J Am Geriatr Soc 2007 55 7 1072 1077 10.1111/j.1532-5415.2007.01217.x 17608881

[B18] Manepalli J Grossberg GT Mueller C Prevalence of delirium and urinary tract infection in a psychogeriatric unit J Geriatr Psychiatry Neurol 1988 36 12 198 202 10.1177/089198879000300404 2073307

[B19] Miller J To treat or not to treat: managing bacteriuria in elderly people CMAJ 2001 164 5 619 620 11258205 PMC80808

[B20] Juthani-Mehta M Drickamer MA Towle V Zhang Y Tinetti ME Quagliarello VJ Nursing home practitioner survey of diagnostic criteria for urinary tract infections J Am Geriatr Soc 2005 53 11 1986 1990 10.1111/j.1532-5415.2005.00470.x 16274383

[B21] Walker S McGeer A Simor AE Armstrong-Evans M Loeb M Why are antibiotics prescribed for asymptomatic bacteriuria in institutionalized elderly people? A qualitative study of physicians' and nurses' perceptions CMAJ 2000 163 3 273 277 10951723 PMC80288

[B22] Nicolle LE Strausbaugh LJ Garibaldi RA Infections and antibiotic resistance in nursing homes Clin Microbiol Rev 1996 100 1 1 17 10.1128/cmr.9.1.1 PMC172878 8665472

[B23] Yoshikawa TT Nicolle LE Norman DC Management of complicated urinary tract infection in older patients J Am Geriatr Soc 1996 22 6 1235 1241 10.1111/j.1532-5415.1996.tb01376.x 8856005

[B24] Takahashi P Trang N Chutka D Evans J Antibiotic prescribing and outcomes following treatment of symptomatic urinary tract infections in older women J Am Med Dir Assoc 2004 5 2 SUPPL S11 S15 14984605 10.1097/00130535-200403001-00006

[B25] Gunnarsson RK Lanke J The predictive value of microbiologic diagnostic tests if asymptomatic carriers are present Stat Med 2002 21 12 1773 1785 10.1002/sim.1119 12111911

[B26] Gunnarsson R Calculator for EPV 2001 http://www.infovoice.se/fou/epv/calc/

[B27] Stamm WE Counts GW Running KR Fihn S Turck M Holmes KK Diagnosis of coliform infection in acutely dysuric women N Engl J Med 1982 307 8 463 468 10.1056/NEJM198208193070802 7099208

[B28] Pfisterer MH Griffiths DJ Schaefer W Resnick NM The effect of age on lower urinary tract function: a study in women J Am Geriatr Soc 2006 54 3 405 412 10.1111/j.1532-5415.2005.00613.x 16551306

[B29] Haidinger G Temml C Schatzl G Brossner C Roehlich M Schmidbauer CP Madersbacher S Risk factors for lower urinary tract symptoms in elderly men. For the Prostate Study Group of the Austrian Society of Urology Eur Urol 2000 37 4 413 420 10.1159/000020162 10765071

[B30] Malmsten UG Milsom I Molander U Norlen LJ Urinary incontinence and lower urinary tract symptoms: an epidemiological study of men aged 45 to 99 years J Urol 1997 158 5 1733 1737 10.1016/S0022-5347(01)64113-2 9334589

